# A Systematic Review of Non-pharmacological Strategies to Reduce the Risk of Violence in Patients With Schizophrenia Spectrum Disorders in Forensic Settings

**DOI:** 10.3389/fpsyt.2021.618860

**Published:** 2021-05-10

**Authors:** Rudolf Slamanig, Andreas Reisegger, Hildegard Winkler, Giovanni de Girolamo, Giuseppe Carrà, Cristina Crocamo, Heiner Fangerau, Inga Markiewicz, Janusz Heitzman, Hans Joachim Salize, Marco Picchioni, Johannes Wancata

**Affiliations:** ^1^Clinical Division of Social Psychiatry, Department of Psychiatry and Psychotherapy, Medical University of Vienna, Vienna, Austria; ^2^Unit of Epidemiological and Evaluation Psychiatry, IRCCS Istituto Centro San Giovanni di Dio Fatebenefratelli, Brescia, Italy; ^3^Department of Medicine and Surgery, University of Milano Bicocca, Milan, Italy; ^4^Department of the History, Philosophy and Ethics of Medicine, Medical Faculty, Heinrich-Heine-University Duesseldorf, Duesseldorf, Germany; ^5^Department of Forensic Psychiatry, Institute of Psychiatry and Neurology, Warsaw, Poland; ^6^Medical Faculty Mannheim, Central Institute of Mental Health, Heidelberg University, Mannheim, Germany; ^7^St Magnus Hospital, Surrey, United Kingdom; ^8^Department of Forensic and Neurodevelopmental Science, Institute of Psychiatry, Psychology and Neuroscience, King's College London, London, United Kingdom

**Keywords:** schizophrenia, forensic psychiatry, non-pharmacological interventions, psychological interventions in forensic settings, violence, systematic review

## Abstract

**Background:** The purpose of this systematic review is to systematically investigate which non-pharmacological interventions are effective in reducing violence risk among patients with schizophrenia spectrum disorders (SSD) in forensic settings.

**Methods:** Six electronic data bases were searched. Two researchers independently screened 6,003 abstracts resulting in 143 potential papers. These were analyzed in detail by two independent researchers yielding 10 articles that could be used.

**Results:** Of the 10 articles, four were non-randomized controlled trials, three were pre-post studies without controls, and one was observational. Only two studies applied a randomized controlled trial design. Cognitive behavioral treatment programs were investigated in three studies. A broad range of other interventions were studied. Often outcome measures were specific to each study and sample sizes were small. Frequently, important methodological information was missing from the papers. It was not possible to carry out a meta-analysis due to the heterogeneity of the study designs and outcome measures.

**Conclusion:** Because of methodological limitations it is difficult to draw firm conclusions about the effectiveness of non-pharmacological interventions to reduce the risk of violence in patents with SSD in forensic psychiatry settings. Studies applying better methods in terms of study design, sample sizes and outcome measures are urgently needed.

## Introduction

One in 100 of the population will develop a schizophrenia spectrum disorder (SSD) during their lifetime. Schizophrenia is a disease with hallucinations, delusions and thought disorders (i.e., positive symptoms). A marked proportion also develops negative symptoms, such as reduced drive or affective blunting (1). While some schizophrenia sufferers recover after some episodes, others have numerous relapses or develop a chronic course. Among those with a chronic course frequently impairments of the cognitive and social skills can be observed. This can lead to the inability of independent housing or to difficulties in working (2, 3).

In addition, several studies reported an increased risk of committing violent crimes among patients with SSD as compared to persons without this disorder. A systematic review (4) demonstrated a clear association between schizophrenia, substance use disorders and violence. They reported an OR of 2.1 for those with schizophrenia only as compared to the general population, with that risk rising when comorbid substance use was also present (OR 8.9). As in non-psychiatric offenders, criminal offenses in patients with SSD are linked profoundly to situational factors. Victims and perpetrators often know each other (5). Even before they develop schizophrenia, a subgroup of patients experienced conduct problems, environmental difficulties and trauma in their childhood (6).

Persons with SSD who had committed violent crimes are usually treated in forensic psychiatric services. Such services usually consist of special high security units providing psychiatric treatment and long-term care in order to limit further harm to the patient as well as the general public. The organization of such forensic services differ largely between countries, e.g., some are stand-alone psychiatric hospitals, while others are part of regular psychiatric inpatient services or are part of prisons (7). As a result, the prevalence and incidence of those treated in these services differ largely between countries (8).

Antipsychotic drugs are effective in improving positive and negative symptoms as well as preventing relapses as had been shown in numerous randomized controlled trials (RCTs) (9, 10). Using national register data Fazel et al. (11) reported that antipsychotics reduce the risk for violent crime among SSD patients, but their data did not give information about patients of forensic settings.

Meta-analyses reported that cognitive-behavioral therapy (CBT) significantly reduces psychotic symptoms in schizophrenia (12). However, a smaller number of studies investigated the effects of CBT or other psychosocial interventions on SSD patients who were aggressive or violent (13). Nevertheless, Haddock et al. (14) reported from a RCT that CBT was effective in violence reduction among SSD patients in general psychiatric services. Some studies investigated the effectiveness of non-pharmacological interventions on violence reduction in other settings such as prisons, but among people without psychiatric diagnoses and reported that cognitive interventions were effective in reducing violence [e.g., (15, 16)]. Other studies among persons with personality disorders found that CBT (17) and Schema therapy (18) were effective in reducing physical aggression or violent attacks.

Rampling et al. (19) performed a systematic review of 23 studies investigating non-pharmacological interventions among severely mentally ill (i.e., with SSD or affective disorders) and reported an improvement in physical aggression after cognitive behavioral interventions for psychoses in general psychiatric settings. A recently published umbrella review of non-pharmacological violence reduction strategies across psychiatric settings identified five reviews, but none in forensic psychiatric services (20).

All these findings indicate that studies of non-pharmacological interventions for violence prevention are scarce for SSD patients in forensic settings. As a result forensic psychiatrists frequently must rely on studies conducted in general psychiatry settings. However, there are differences between patients with SSD in general psychiatric and forensic settings. Forensic patients tend to have a more difficult chronic illness course, higher numbers of short-term admissions before their index violence, higher rates of comorbid substance use disorders, lower treatment compliance and lower levels of insight into both their mental disorder and the risk of violence (21, 22). Forensic patients also have more persistent positive psychotic symptoms and higher levels of cognitive impairment (23). Thus, it remains unclear if the non-pharmacological interventions developed and evaluated in general psychiatry are effective in forensic psychiatry, too.

### Aims

To date there has been no systematic reviews of non-pharmacological interventions for reducing the risk for violence in people with SSD within forensic settings (24). Considering that a lot of financial and clinical resources are used for these services it seems to be an urgent necessity to provide such a systematic review of available research. Thus, we decided to conduct a systematic review of studies among forensic patients with SSD, having evaluated non pharmacological interventions without any limitations in order to prevent the risk of violence. Since we expected a rather small number of studies in this area we did not limit our search regarding study designs or comparison groups.

## Methods

This systematic review was conducted according to the Preferred Reporting Items for Systematic reviews and Meta-Analyses guidelines [PRISMA; (25)]. The protocol was registered with the PROSPERO International Prospective Register of Systematic Reviews (registration number CRD42019146381).

We conducted a systematic literature search of Medline, PsycINFO, and Psyndex Lit & AV via Ovid search engine, CINAHL via EBSCOhost, Scopus, Web of Science (Core Collection) and EMBASE. The search strategy is listed in the Supplementary Material Table S1. We decided to adopt an explicitly broad search query in order to include the widest variety of possible interventions. Since the demarcation between violence and aggression is not always consistent, we included both terms into our search pattern. Some authors would put the term “violence” on the far end of the spectrum of aggressive behavior: i.e., representing actions with the purpose to inflict severe physical harm, as injury or death, on another person (26).

**Figure 1:**
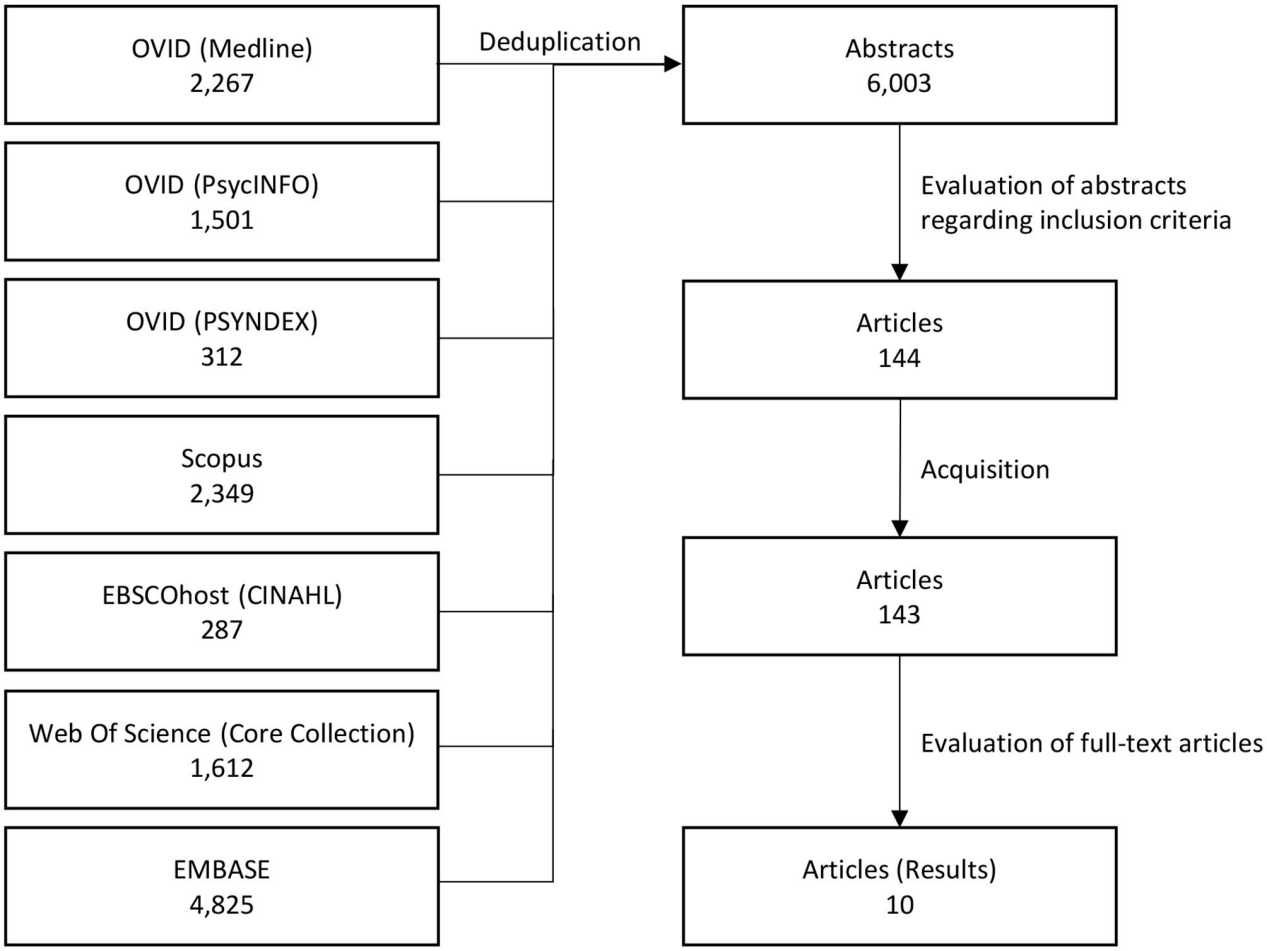
Flow diagram.

The EMBASE search was performed on November 15th, 2019. All other searches were performed on November 12th, 2019. After duplicates were removed using search engine tools and the EndNote deduplication function, 6,003 articles remained (Figure 1). Two out of three researchers (RS, AR, HW) then independently screened the abstracts according to our inclusion criteria:

- participants aged 18 years or older;
- participants suffering from SSD;
- non-pharmacological interventions;
- randomized and non-randomized controlled trials as well as observational studies performed in forensic psychiatric in- or outpatient settings;
- outcome measure: violent or aggressive behavior;
- published in peer reviewed journal;
- published in 1990 or later;
- Published in English.

Of the remaining 144 papers, one paper could not be sourced in either electronic or paper version. The 143 available texts underwent an in-depth analysis by two researchers (RS, AR) against the inclusion criteria and extracted suitable outcomes from relevant papers. Publications were excluded because:

- 2 texts were duplicates.
- 11 texts covered other topics (e.g., genetic risk factors for violence).
- 32 texts were not original research papers, for example reviews, meta-analyses, or abstracts.
- 27 articles either did not investigate non-pharmacological interventions or did not measure the effect of the intervention.
- 14 articles contained exclusively qualitative measures,
- 12 articles reported the findings of studies exclusively outside forensic settings.
- 21 articles had insufficient data on violence outcomes
- 12 articles covered either other diagnoses, excluded SSDs or did not discriminate across diagnostic groups at all
- 2 articles failed to meet the age criterion.

The raters agreed perfectly on the exclusions [Cohen's kappa = 1 (27)]. This left only 10 articles that qualified for the systematic evaluation. For each eligible article and intervention, the most direct violence measures were extracted and evaluated. A structured sheet was used for data extraction from each study (i.e., year of publication; country; inclusion criteria; setting; sample size; tested non-pharmacological treatments; study duration; main findings). Two researchers independently extracted the data, and any differences were resolved by consensus with other co-authors.

### Quality of Evidence

The quality of evidence was assessed using the GRADE method (28). Outcomes were rated individually by two researchers and disagreements settled by consensus. The final rating included estimates of the

- risk of bias
- inconsistency
- indirectness
- imprecision
- and publication bias.

The overall quality of evidence for an outcome can be rated between very low and high, starting at high for RCTs and low for observational studies.

Due to heterogeneity in both outcome measures and design it was impossible to compare the results of the included studies from a statistical point of view. Where appropriate, standardized effect sizes (Cohen's d) were estimated.

## Results

The final analysis yielded 10 studies that included a total of 1,551 subjects, of whom <1% were female. Five studies were performed in the United Kingdom, two in the USA, and one each in Germany, New Zealand and in the Netherlands (Table 1).

**Table 1:** Description of the papers included.

**References**	**Lang**.	**Country**	**Setting**	**Intervention**	**Measure**	**Design**
Ahmed et al. (29)	EN	USA	Hospital with forensic and mental health units	Cognitive remediation group (+ med. Treatment); three 60-min sessions (50 min computerized cognitive activities + 10 min bridging group discussion) per week	OAS: physical aggression	RCT
Carmel et al. (30)	EN	USA	Maximum security forensic hospital	Patients per physician/psychiatrist	Number of incidents of patient aggression (Atascadero Monthly Massed Special Incident Data Base)	Observational study
Cullen et al. (31)	EN	UK	Six medium secure forensic hospitals in the United Kingdom	Reasoning and Rehabilitation (RandR) vs. TAU; 36 sessions á 2 h, 2–3/week (completion at 30 sessions)	Violent incidents as any physically violent behavior (Mac Arthur Community Violence Instrument)	RCT
Daffern et al. (32)	EN	UK	Detained patients under UK Mental Health Act	Life Minus Violence-Enhanced (LMV-E); > 125 treatment sessions, total ~300 h; group and individual setting	HCR-20; no. of acts of aggression (verbal, physical aggression, deliberate property damage)	Non-randomized controlled trial
Davies et al. (33)	EN	UK	Medium secure mental health forensic service	Positive Behavioral Support (PBS); comprehensive plan for support of individual needs and interests	Checklist of Challenging Behavior (CBC)—aggression frequency and aggression severity subscales	Non-randomized controlled trial
Fluttert et al. (34)	EN	Belgium, Nether-lands, Norway	16 wards of a maximum security forensic hospital	Early Recognition Method (ERM); weekly assessment	Number of seclusions, severity of inpatient incidents	Pre-post study, no controls
Lohner et al. (35)	DE	Germany	Adult patients within the penal system with acute need of psychiatric treatment	Integrated medical, psychotherapeutical, sociotherapeutical treatment program; daily group therapy	Estimated risk of harm to others (by treating psychiatrist)	Non-randomized controlled trial
Reiss et al. (36)	EN	UK	High security ('special') hospital.	Theater project (drama therapy + CBT concepts); 5 days: 2 plays, series of workshops, final “challenge.”	Custom 25-items, 5-point Likert-scale; State-Trait Anger Expression Inventory (STAXI AX/out dimension)	Pre-post study, no controls
Sistig et al. (37)	EN	New Zealand	Forensic inpatient service	Mindful yoga; 8 weekly 60 min classes, 30 min guided homework, 2-page A4 poster	CORE-OM subscale “risk to self and others.”	Pre-post study, no controls
Yip et al. (39)	EN	UK	Detainment under the U.K. Mental Health Act	Reasoning and Rehabilitation Mental Health Programme (R&R2MHP) vs. TAU; 16 session, 1/week, 90 min; “completion”: 80% attendance	Maudsley Violence Questionnaire (MVQ)/acceptance-of-violence subscale, Novaco Anger Scale—Provocation Inventory (NAS-PI)	Non-randomized controlled trial

The evaluated interventions broadly speaking broke down into neurocognitive training (one study), cognitive-behavioral therapy (three studies), and other non-pharmacological interventions (six studies). Five studies investigated the effects of group interventions, two of individual interventions and another two combinations of group and individual interventions. One study analyzed the influence of staff-patient-ratio. In line with the vast differences in these treatment approaches, there was also an enormous variation in both the duration of the intervention programs (between 5 days and 12 months) and the follow up observation periods (up to 56 months). Three were non-randomized controlled trials, three were pre-post studies without controls, and one was merely observational. Only two studies applied a RCT design. Seven studies used scales or questionnaires to assess violence or aggression, and three studies counted incidents of violence or of seclusions.

### Neurocognitive Training

Ahmed et al. (29) performed an unblinded RCT to examine the effects of a cognitive remediation program over 20 weeks. Eligible patients who had been violent were randomized to either the intervention or an active control group (Table 2). A sample of 42 patients (4 female) with a diagnosis of schizophrenia (*N* = 27) or schizoaffective disorder (*N* = 15) from both forensic and general adult settings received 50 h of computer-based cognitive remediation therapy. Results were compared with a control group, who followed a comparable program of 3 weekly computer game sessions. In the combined general psychiatric and forensic sample patients in the intervention group were less violent at follow up as measured by the Overt Aggression Scale [OAS (40)] over the 20 week follow up period. Due to a small sample size, without a power analysis, the quality of evidence was considered moderate.

**Table 2:** Effects of non-pharmacological trials (Treatment as usual = TAU).

**References**	**Intervention**	**Outcome**	**Control condition (TAU)**	**Intervention condition**	**Absolute effect**	**Relative effect**	**Effect size (Cohen's d)**	**Persons in control group (studies)**	**Persons in intervention group (studies)**	**Quality of the evidence (GRADE)**
Ahmed et al. (29)	Cognitive Remediation Group (+ TAU) vs. TAU	OAS: physical aggression score	Mean = 0.61; SD = 1.08	Mean = 0.17; SD = 0.49	MD = 0.440; SE = 0.185		0.54	36 (1 study)	42 (1 study)	⊕⊕⊕○ Moderate^a^^,^^b^^,^^c^
Carmel et al. (30)	Number of present physicians	Number of incidents of patient aggression				*r* = 0.38 (p/phys)	0.82		973 (1 study)	⊕○○○ Very Low^g^
	Number of present psychiatrists	Number of incidents of patient aggression				*r* = 0.35 (p/psy)	0.74		973 (1 study)	⊕○○○ Very Low^g^
Cullen et al. (31)	Reasoning and Rehabilitation (R&R) vs. TAU	Violent incidents as any physically violent behavior (Mac Arthur Community Violence Instrument)				IRR: 0.52 [0.23, 1.15] (end of treatment); 0.86 [0.44, 1.66] (12 mo follow-up)		40 (1 study)	44 (1 study)	⊕⊕⊕⊕ High^b^^,^^h^
Daffern et al. (32)	Effect of Life Minus Violence-Enhanced (LMV-E) program on estimated risk for violence	HCR-20	Mean = 17.5; SD = 3.86	Mean = 25.28; SD = 5.8	MD = −8.13; SE = 1.463		−1.575	42 (1 study)	33 (1 study)	⊕○○○ Very Low^a^^,^^d^^,^^e^^,^^i^
Davies et al. (33)	Positive Behavioral Support (PBS)	“Aggression frequency” CBC (adapted) subscale	Mean = 7.94 (range 0–40)	Mean = 2.35 (range 0–9)	MD = 5.2; SE = n/a			17 (1 study)	17 (1 study)	⊕○○○ Very Low^a^^,^^d^^,^^e^^,^^g^
	Positive Behavioral Support (PBS)	“Aggression severity” CBC (adapted) subscale	Mean = 3.24 (range 0–17)	Mean = 0.88 (range 0–5)	MD = 2.36; SE = n/a			17 (1 study)	17 (1 study)	⊕○○○ Very Low^a^^,^^d^^,^^e^^,^^g^
Fluttert et al. (34)	Early Recognition Method for psychosis	Number of seclusions/patient/month	Mean = 0.09; SD = n/a	Mean = 0.04; SD = n/a	MD = 0.05 (frequency); SE = n/a		0.43		86 (1 study)	⊕○○○ Very Low^a^^,^^e^
	Early Recognition Method for psychosis	Severity of incidents. SOAS-R × seclusions/patient/month	Mean = 0.8; SD = n/a	Mean = 0.41; SD = n/a	MD = 0.35 (severity); SE = n/a		0.39		86 (1 study)	⊕○○○ Very Low^a^^,^^e^
Lohner et al. (35)	Integrated medical, psychotherapeutic, sociotherapeutic treatment program	Estimated risk of harm to others (by treating psychiatrist)				RR = 1.13		n/a	124 (1 study)	⊕○○○ Very Low^a^^,^^b^^,^^i^
Reiss et al. (36)	Therapeutic theater project	Customized questionnaire, scale “how angry”	Mean = 35.2; SD = 14.3	Mean = 22; SD = 12.2	MD = 13.2; SE = 5.426		0.99		12 (1 study)	⊕○○○ Very Low^a^^,^^d^^,^^e^^,^^j^
	Therapeutic theater project	Customized questionnaire, scale “how react”	Mean = 16.3; SD = 12.4	Mean = 5.2; SD = 6.2	MD = 11.1; SE = 4.002		1.132		12 (1 study)	⊕○○○ Very Low^a^^,^^d^^,^^e^^,^^j^
	Therapeutic theater project	STAXI, AX/out	Mean = 16.; SD = 2.1	mean = 14; SD = 3.6	MD = 2; SE = 1.203		0.679		12 (1 study)	⊕○○○ Very Low^a^^,^^d^^,^^e^^,^^j^
Sistig et al. (37)	Mindful Yoga	“Risk to self and others” CORE-OM subscale	Mean = 1.85; SD = 3.65	Mean = 13.5; SD = 2.64	MD = 0.5; SE = 0.852		0.163		26 (1 study)	⊕○○○ Very Low ^a^^,^^d^^,^^e^^,^^k^
Yip et al. (39)	Reasoning and Rehabilitation Mental Health Programme (R&R2MHP) vs. TAU	Maudsley Violence Questionnaire (MVQ)/acceptance of violence	Mean = 8.48; SD = 3.97	Mean = 6.60; SD = 3.12	MVQ/acceptance: MD = 2.450; SE = 0.965		0.53	29 (1 study)	30 (1 study)	⊕○○○ Very Low^a^^,^^d^^,^^e^^,^^f^
	Reasoning and Rehabilitation Mental Health Programme (R&R2MHP) vs. TAU	Novaco Anger Scale—Provocation Inventory (NAS-PI)/Behavior domain	Mean = 26.14; SD = 7.30	Mean = 25.20; SD = 5.91	NAS-PI: MD = 0.130; SE = 5.109		0.14	29 (1 study)	30 (1 study)	⊕○○○ Very Low^a^^,^^d^^,^^e^^,^^f^

a *Small sample size*.

b *Limited allocation concealment and blinding, probably no limitation to validity*.

c *Mixed general and forensic sample, but statistically checked for comparability*.

d *No or insufficient allocation concealment*.

e *No or insufficient blinding*.

f *Unknown number of participants with inadequate diagnosis*.

g *No exact information about patient diagnoses (but “majority schizophrenia”)*.

h *Single study, small sample size, but power analysis*.

i *Outcome data incomplete*.

j *Majority with inadequate diagnosis*.

k *Around 25% with inadequate diagnosis*.

### Cognitive-Behavioral Treatment Programs

Cullen et al. (31) performed an RCT to examine the impact of a Reasoning and Rehabilitation (R&R) program on the reduction of violence and antisocial behavior in a forensic psychiatric population. R&R is a highly structured manualized cognitive-behavioral intervention (41). All participants who attended at least 30 sessions were included in this study. The sample included patients with schizophrenia, schizoaffective disorder, bipolar disorder or any other psychotic disorder. Participants were randomized to the intervention or a passive control group. The effectiveness of the intervention was measured by the number of violent or antisocial incidents during treatment and at 12 months. Of the 44 initial participants, more than half failed to complete 30-sessions (52.3%). There were no significant differences in violence incident rates between the two groups either at the end of the intervention (Incidence Rate Ratio = IRR: 0.52 [0.23–1.15]; *p* = 0.11), or at the 12-month follow-up (IRR: 0.86 [0.44–1.66]; *p* = 0.65). The authors conducted a power analysis for estimating the necessary sample size. We considered this a high quality study.

Yip et al. (39) enrolled 30 adult male inpatients in a high-secure hospital in a Reasoning and Rehabilitation program adapted for offenders with severe mental illness (R&R2 MHP). Around 80% of the participants completed the program. Allocation to the intervention group was determined by order of referral, and the sample was compared to a control group of 29 forensic male inpatients placed on a waiting list, undergoing treatment as usual. For the purpose of this review, violence outcome data were extracted from subscales of the Maudsley Violence Questionnaire [MVQ; (42)], and the Novaco Anger Scale—Provocation Inventory: Reaction to Provocation/Personal Affect Questionnaire [NAS-PI (43)]. While the NAS-PI showed no statistically significant differences, the “acceptance of violence” subscale from the MVQ produced a significant moderate reduction in violence (Cohen's d = 0.53; *p* < 0.01). The study included an unspecified number of patients with a primary diagnosis of an affective disorder. The lack of randomization and blinding lead us to consider the study as having a very low quality.

A recent study involving a sample of forensic patients with a history of violence and diagnoses of paranoid schizophrenia (*n* = 19) and paranoid schizophrenia as well as antisocial personality disorder (*n* = 14), tested the effect of the Life Minus Violence-Enhanced (LMV-E) program on violence and aggressive behavior (32). The study was conducted in a high security mental health hospital in the UK. A control group of potentially eligible candidates who did not participate in the program was included. The violence outcome was the HCR-20 total score at the end of the intervention. Although violence risk reduced in both groups, surprisingly the control group showed a significantly greater reduction in violence risk (*p* < 0.001). Due to the lack of randomization and blinding, as well as a small sample size, we rated this as a study of very low quality.

### Other Interventions

Lohner et al. (35) analyzed the impact of an integrated treatment program in a forensic hospital in Germany, which consisted of pharmacological treatment with behavioral and educational elements. Structured educational groups focused on coping strategies and cooperation. Other elements involved occupational therapy, art therapy, sports therapy, cognitive training, and psychodynamic therapy. One hundred and twenty four male inpatients in one of the treatment program wards were compared to patients in other forensic psychiatric wards at the same hospital. Patients ward allocation was determined by bed availability. All patients had a primary ICD-10 F2 diagnosis. There was no significant difference for the staff estimated risk of causing harm to others at hospital discharge (*p* > 0.05). This paper provided no information on the method of staff risk assessment. Together with the limits concerning randomization (allocation to each ward potentially influenced by medical indication and individual capacity), the quality of evidence has to be considered very low.

Using a pre-post-design Fluttert et al. (34) evaluated the effect of an Early Recognition Method (ERM) in 16 wards of a maximum security forensic hospital in the Netherlands. ERM aims to improve patients ability to perceive and communicate the need for preventive actions. ERM was integrated into pre-existing scheduled interactions between patients and staff, and required ~30 min per week. One hundred and sixty eight male patients of whom 90 had a schizophrenia diagnosis were included. The number of incidents before and after treatment was compared. The number of seclusions and the severity of violent incidents significantly decreased (*p* < 0.05) after the implementation of the ERM, in both the wider sample and the schizophrenia subsample. Due to the lack of a control group, lack of randomization and blinding, this study is considered to have a very low quality.

Davies et al. (33) investigated the impact of Positive Behavioral Support (PBS) plans in UK medium secure forensic hospitals. After a functional assessment of each participant's violent behavior, measures were planned cooperatively between patients and ward staff to address violence triggering or supporting factors. Twenty two patients with a PBS-plan (18% female, 59% with a SSD diagnosis) were compared to 17 patients on a waiting list for the same treatment. Group allocation was clinical decision. Violence outcome was assessed using the Checklist of Challenging Behavior [CBC (44)]. Compared to the control group, the frequency of violence and the management difficulty at 12 months-follow up was significantly (*p* < 0.05) lower in the PBS group. Methodological limitations such as the lack of randomization and rater blinding indicated that the quality of evidence was very low.

Carmel et al. (30) looked at the relationship between the number of medical staff and violent incidents in a maximum-security forensic hospital in California, USA. In that 973-bed institution, over a 56-month period, all 13,209 special incident reports including 7,389 incidents of patient aggression/violence were identified, as was the number of medical staff present at the hopital at the time for these incidents. The number of incidents with physical aggression was negatively correlated with both the number of patients per general physician (*r* = 0.38; *p* < 0.005) and the number of patients per psychiatrist (*r* = 0.35; *p* < 0.01). Non-violent episodes of dangerous behavior were also related to the number of patients per psychiatrist. This observational study offered no information about the diagnoses in the sample other than that the “majority” had SSD. On that basis we assessed the study as having a very low quality.

Reiss et al. (36) evaluated the effects of a therapeutic theater project on anger in forensic psychiatric patients. This study was included because anger strongly predicts an aggressive predisposition (45). A total of 12 male patients (5 with a SSD) at the young persons' unit (age 18–30 years) at a high security hospital in the United Kingdom, took part in a 5-day theater project, following drama therapy and CBT principles. Two plays were staged after a series of workshops. Self-report of aggressiveness was assessed at baseline, after the 5-day project, and at 3 months follow-up, using a custom 25-item anger inventory, and the State-Trait Anger Expression Inventory [STAXI (46)]. The subscales “how angry” (affective response) and “how react” (behavioral response), and the “anger-out” (anger expressed toward other people) subscale of the 25-item anger inventory showed significant improvements both after the intervention and at later follow-up, while the STAXI showed no statistically significant differences at either time point. Due to the lack of blinding and randomization and the small sample size, the quality of evidence was rated as very low.

Sistig et al. (37) evaluated the impact of a specially adapted yoga program on stress and anxiety in patients in a forensic psychiatric institution in New Zealand. The “mindful yoga” program consisted of 8 weekly classes of 60 min, 30 min of guided homework, and a 2-page A4 poster. Of the 32 initial participants, 7 of whom were female and 77% had a diagnosis of SSD, 26 completed the program. The sub-score “risk to self and others” of the Clinical Outcomes in Routine Evaluation—Outcome Measure (CORE-OM) indicating the staff perception of the patients' risk of violent behavior showed no statistically significant effect. The study had no control group, was unblinded and gave no information about the allocation procedure so the quality of evidence is very low.

## Discussion

This is the first systematic review reporting the effects of non-pharmacological interventions on the risk of violent behavior among SSD patients in forensic psychiatry. Overall, despite a very comprehensive search strategy we found only 10 studies on this topic. This matches with the recently published paper by Howner et al. (38) who reported that they independent of type of mental diagnosis treated in forensic psychiatry found no systematic review with a low risk of bias, and only four systematic reviews having a moderate risk of bias. Most of the original studies included into these four systematic reviews had a high risk of bias prohibiting quantitative meta-analyses. None of these systematic reviews had a focus on SSD. This indicates a huge lack of research in this area.

### Study Design and Analyses

The studies used a wide range of research designs from RCTs, non-randomized controlled trials, pre-post comparisons without controls and observational studies. Studies without controls can definitely not be used to establish whether the interventions yielded any beneficial effects. Even studies using a control group can be biased, if the control group differs in key characteristics from the intervention group. For example, Daffern et al. (32) reported better results in the control group than the intervention group without establishing if there were any differences between the two groups. That raises the possibility that confounding variables might explain the results. Of course, the results could be influenced by other interventions such as psychotropic treatment, staff-patient ratio, severity of psychiatric symptoms or illness history. Psychotropic medicines remain the key intervention in the treatment of most patients with SSDs, pharmacological regimes will often differ between clinical teams, wards and institutions. RCTs provide the best approach to minimize the problem of confounding. However, in real life it is often impossible to use RCTs in forensic clinical settings due to various practical reasons. If randomization is not feasible, researchers should at least report relevant baseline data from study groups, which might influence the effect of treatment and attempt to match groups as closely as possible on such factors (47). We note that for very novel and innovative interventions small scale studies may yield important information of a therapeutic effect, that will then help to justify plans for more sophisticated and costly studies (48).

Both studies that applied an RCT design (29, 31) used an intention-to-treat approach for their analyses. This approach takes into account all subjects included in the study, even those who dropped out. That, while telling us something about the tolerability of the intervention, is at risk of leading to an underestimation of the effectiveness of the intervention. In contrast, analyzing only those who completed the study might be biased by including only the most motivated or responsive patients. Papalia et al. (47) suggested that future studies should report on both the results of the intention-to-treat as well as completers samples.

The sample size of most of the studies that we identified was small. That finding might partly explain the large number of non-significant findings among these studies. In most papers the authors did not report a pre-study power analysis. Thus, they could not plan their studies based on this kind of information, which, in turn, makes it difficult for readers to decide how to interpret negative results. Although we focused on SSDs, frequently subjects with other diagnoses were included in these studies. The data were often not reported separately for patients with SSD and other diagnoses. Furthermore, most studies did not use standardized diagnostic instruments, such as the SCID, to confirm diagnoses or illness severity scales.

### Assessments

Outcome measures used to quantify the level or risk of violence varied considerably between the studies. While some authors (29) used standardized and validated scales such as the Overt Aggression Scale (40), others simply counted the number of seclusions or aggressive incidents recorded in the patients' hospital files (30, 34). The nature of what constituted aggression and violence also varied. This is particularly true for lower level violent incidents. In some studies the definition of aggression and violence may have been influenced by legal or clinical considerations thus hindering comparability between studies. Similarly, national definitions, legal and clinical rules and considerations may influence how often seclusions are used rather than any study intervention. There are a range of validated instruments to record and quantify violence for clinical and research purposes in mental health settings (49). Where violence is recorded and how that data is accessed is also important. Using multiple sources of information, such as self-report, clinical assessments and patient files will yield the most comprehensive information.

It is also clear, with regard to study outcome, that the duration of the follow-up varied considerably. We assumed that the effect of any non-pharmacological intervention will persist at least for some weeks (29) and possibly for longer (31). Of course, longer follow up periods allow more time for violent incidents to occur, though these will be matched between study groups. However, given that the aim of forensic services in general is to produce long term violence risk reduction and allow discharge to less restrictive settings, better designed studies with longer term follow up is needed.

### Findings of This Review

The range of interventions studied was very broad from training in the early recognition of symptoms, cognitive remediation therapy to therapeutic theater and yoga. CBT was the most frequently investigated type of intervention (31, 32, 39). This matches with the finding of Rampling et al. (19) who reported that several studies exist which reported positive effects of CBT in reducing physical aggression among severely mentally ill including SSD patients in general psychiatry. Similar to our review Darmedru et al. (50) reported that in general psychiatry cognitive remediation was effective in the reduction of aggressive behaviors and physical assaults in schizophrenia.

The studies included group programs [e.g., (29)], individual interventions [e.g., (34)] and combinations of group and individual interventions [e.g., (32)]. Papalia et al. (47) reported from their review of psychological treatments that group-based interventions were associated with greater reductions in violent recidivism relative to treatments that used individual delivery only. Due to the heterogeneity of study designs and interventions in our review, we cannot verify if this holds true for patients with SSD.

Interventions ranged widely in their demands and duration. Some consisted of at least 125 treatment sessions (32) while others had only 8 sessions (37). This wide span place hugely different demands on clinical budgets and staffing levels and training. These considerations will influence what interventions might be implemented in clinical settings, balanced against the evidence of clinical effectiveness.

There was an interesting finding in the observed changes in violent incidents linked to the ratio between medical staff and forensic patients. Violence was less when there were more psychiatric or general medical personnel. This relationship might seem obvious, though the underlying mechanisms are unclear. More medical staff members could lead to more time per patient for treatment planning, evaluation and risk assessment, therefore potentially improving outcomes. On the other hand, it is plausible that the mere presence of staff produces a sense of security and therefore has a preventive effect on aggressive and violent behavior. However, staff numbers can also be having effects by implementing interventions such as the “Early Recognition Method” or preventive strategies like “Positive Behavioral Support”-plans. Reliable violence risk assessment and management takes time and effort, safe staffing levels need to be available.

### Limitations

Despite the fact that we used a very comprehensive search strategy we found only a very small number of studies that attempted to provide evidence for the impact of non-pharmacological treatments to patients with SSDs aiming to reduce violence in forensic psychiatry settings. This is striking given the number of patients that could directly benefit but also the wider implications for society. It is also striking given that many forensic services invest so heavily in such therapies without a clear evidence base. Despite applying a very comprehensive search strategy, for practical reasons we excluded some specific forms of aggression such as child abuse, school violence or terrorism. We cannot rule out that we might have overlooked a small number of papers. We limited our search strategy to articles published since 1990 because the forensic psychiatry field has changed so radically over the intervening three decades. Of course, this lack of data could be considered a limitation of this review, but we think that it is of itself a very relevant finding. Forensic psychiatry services invest huge amounts of time and resources in non-pharmacological therapies, yet there is a very poor evidence base to support that expenditure. Furthermore, in some jurisdictions, patients remain detained in forensic hospitals until they engage in such violence reduction treatment and until the treatment is completed. This could be considered unethical if the treatment cannot be shown to offer benefit to the patient or other people.

The second main finding was that although we included only articles published in peer-reviewed journals hoping to yield studies with an adequate level of methodological rigor, the results were very disappointing. In general, even in the published literature the quality of evidence was poor to very poor. There is therefore a pressing and urgent need to conduct methodologically robust studies to test what works, expand what does, and stop what does not.

Since we did not search for book chapters, congress abstracts or unpublished studies, we might have overlooked some studies. Nevertheless, we expect that the large majority of sophisticated studies would have been published in peer-reviewed journals. We did not search for studies published in other languages than English. Thus, we cannot exclude that we have missed a small number of studies.

## Conclusions

Because of the methodological limitations of the studies in our review, it is not possible to draw any firm conclusions about the effectiveness of non-pharmacological interventions to reduce the risk of violence in patients with SSD in forensic settings. Two papers (29, 31) reported of RCTs showing that more ambitious study designs can be realized even in forensic settings with SSD patients. The methodological limitations of those two projects (e.g., mixed samples, diagnostic heterogeneity) could be resolved in future studies. Other review papers reporting on other studies in forensic and correctional settings confirm this conclusion (47, 48).

What should be done in everyday work with SSD patients in forensic services until we have more sophisticated studies? At the moment, we must rely on findings from clinical psychiatry showing that some psychological interventions are effective to reduce violence among patients with SSD (50). Findings from reviews showed that some psychological interventions, mainly cognitive-behavioral, are effective for reducing violence. Other studies among offenders without psychiatric diagnoses support this idea (47, 51). Of course, there are important differences between SSD patients in general psychiatry and in forensic settings, but at the moment forensic psychiatrists must rely to a large extent on research conducted in general psychiatry settings.

This systematic review clearly shows that high quality research in this area is urgently needed. It is important that future studies plan sample sizes that are sufficiently powered to confidently address the research questions. In addition, studies should use standardized diagnostic procedures for SSD, use clear definitions of violence which can easily be compared with other studies and are clinically relevant (e.g., number of violent attacks against hospital staff or other people, criminal violence or incarceration). The use of standardized and validated assessment instruments can improve the description of forensic samples. The consequences of lacking research in this area is currently that people are detained against their wishes in forensic hospitals, and often treated against their wishes using interventions which frequently lack high-quality evidence regarding their effectiveness. This raises serious ethical concerns.

## Data Availability Statement

The original contributions presented in the study are included in the article/Supplementary Material, further inquiries can be directed to the corresponding author/s.

## Author Contributions

AR and RS planned the literature search, selected the abstracts, extracted data from original papers, and wrote the original draft. HW selected the abstracts and reviewed the manuscript. GdG and GC planned the literature search, submitted the grant, and reviewed the manuscript. CC and IM commented the methods and reviewed the manuscript. HF and JH prepared the grant and reviewed the manuscript. HS and MP made suggestions for literature searches and reviewed the manuscript. GdG and GC planned the literature search, prepared the grant, and reviewed the manuscript. All authors contributed to the article and approved the submitted version.

## Conflict of Interest

The authors declare that the research was conducted in the absence of any commercial or financial relationships that could be construed as a potential conflict of interest.
