# Supplementary material for: A Systematic Review of Non-pharmacological Strategies to Reduce the Risk of Violence in Patients With Schizophrenia Spectrum Disorders in Forensic Settings
Source: Front Psychiatry. 2021 May 10;12:618860. doi: 10.3389/fpsyt.2021.618860 (PMC8141623; doi:10.3389/fpsyt.2021.618860)
Supplement: Supplementary file 1 [file Table_1.docx]

**Supplementary material to**

**A systematic review of non-pharmacological strategies to reduce the risk of violence in patients with schizophrenia spectrum disorders in forensic settings.**

**Table S1:** Search terms used for electronic databases

| OVID - MEDLINE: 2,267 |
| --- |
| (exp "aggression"/ OR exp "self-injurious behavior"/ OR (exp "violence"/ NOT (exp "domestic violence"/ OR exp "gender-based violence"/ OR exp "intimate partner violence"/ OR exp "terrorism"/)) OR ("violen*" OR "*aggressi*" OR "agitat*" OR "assault*" OR "homicid*" OR ("*self" ADJ2 ("harm*" OR "injur*" OR "mutilat*" OR "destruct*") OR "*suicid*")).mp) AND (exp "schizophrenia spectrum and other psychotic disorders"/ OR ("severe" ADJ3 ("mental" OR "psychiatric") ADJ3 ("disorder*" OR "illness*" OR "condition*" OR "disease*" OR "diagnos*")).mp OR ("psychosis" OR "psychot*" OR "schizophreni*").mp) AND ((exp "prisons"/ NOT exp "concentration camps"/) OR (exp "forensic psychiatry"/ NOT (exp "confidentiality"/ OR exp "insanity defense"/)) OR (exp "prisoners"/ NOT exp "prisoners of war"/) OR ("forensi*" OR "*prison*" OR "incarcerat*" OR "jail*" OR "peniten*" OR "convict*" OR "inmate*" OR "detention*" OR "arrest*" OR "detain*").mp) |
| Filter: ≥ 1990 |
| OVID - PsycINFO: 1,501 / PSYNDEXplus: 312 |
| (exp "aggressive behavior"/ OR exp "self-destructive behavior"/ OR (exp "violence"/ NOT (exp "domestic violence"/ OR exp "school violence"/ OR exp "intimate partner violence"/)) OR ("violen*" OR "*aggressi*" OR "agitat*" OR "assault*" OR "homicid*" OR ("*self" ADJ2 ("harm*" OR "injur*" OR "mutilat*" OR "destruct*") OR "*suicid*")).mp) AND (exp "psychosis"/ OR ("severe" ADJ3 ("mental" OR "psychiatric") ADJ3 ("disorder*" OR "illness*" OR "condition*" OR "disease*" OR "diagnos*")).mp OR ("psychosis" OR "psychot*" OR "schizophreni*").mp) AND (exp "correctional institutions"/ OR exp "forensic psychiatry"/ OR exp "incarceration"/ OR (exp "prisoners"/ NOT exp "prisoners of war"/) OR ("forensi*" OR "*prison*" OR "incarcerat*" OR "jail*" OR "peniten*" OR "convict*" OR "inmate*" OR "detention*" OR "arrest*" OR "detain*").mp) |
| Filter: ≥ 1990; "peer-reviewed journal” |
| SCOPUS: 2,349 |
| TITLE-ABS-KEY (("violen*" OR "*aggressi*" OR "agitat*" OR "assault*" OR "homicid*" OR ("*self" W/2 ("harm*" OR "injur*" OR "mutilat*" OR "destruct*") OR "*suicid*")) AND (("severe" W/3 ("mental" OR "psychiatric") W/3 ("disorder*" OR "illness*" OR "condition*" OR "disease*" OR "diagnos*")) OR ("psychosis" OR "psychot*" OR "schizophreni*")) AND ("forensi*" OR "*prison*" OR "incarcerat*" OR "jail*" OR "peniten*" OR "convict*" OR "inmate*" OR "detention*" OR "arrest*" OR "detain*")) AND PUBYEAR > 1989 |
| Filter: "Article" |
| CINAHL - EBSCOhost: 287 |
| (((MH "Aggression+") NOT ((MH "Child Abuse") OR (MH "Elder Abuse") OR (MH "Stalking") OR (MH "Student Abuse") OR (MH "Verbal Abuse") OR (MH "Community Violence") OR (MH "Dating Violence") OR (MH "Child Abuse, Sexual") OR (MH "Munchausen Syndrome By Proxy") OR (MH "Domestic Violence+") OR (MH "Intimate Partner Violence") OR (MH "Child to Parent Abuse") OR (MH "School Violence") OR (MH "Sibling Violence"))) OR (MH "Self-Injurious Behavior") OR (MH "Suicide+") OR ("violen*" OR "*aggressi*" OR "agitat*" OR "assault*" OR "homicid*" OR ("*self" N2 ("harm*" OR "injur*" OR "mutilat*" OR "destruct*") OR "*suicid*"))) AND ((MH "Psychotic Disorders+") OR ("severe" N3 ("mental" OR "psychiatric") N3 ("disorder*" OR "illness*" OR "condition*" OR "disease*" OR "diagnos*")) OR ("psychosis" OR "psychot*" OR "schizophreni*")) AND ((MH "Prisoners") OR (MH "Correctional Facilities") OR ((MH "Forensic Psychiatry+") NOT (MH "Insanity Defense")) OR ("forensi*" OR "*prison*" OR "incarcerat*" OR "jail*" OR "peniten*" OR "convict*" OR "inmate*" OR "detention*" OR "arrest*" OR "detain*")) |
| Filter: "Wissenschaftliche Zeitschriften" (i.e. Scientific journals) |
| Web Of Science (Core Collection) 1,612 |
| TS=(("violen*" OR "*aggressi*" OR "agitat*" OR "assault*" OR "homicid*" OR ("*self" NEAR/2 ("harm*" OR "injur*" OR "mutilat*" OR "destruct*")) OR "*suicid*") AND (("severe" NEAR/3 ("mental" OR "psychiatric") NEAR/3 ("disorder*" OR "illness*" OR "condition*" OR "disease*" OR "diagnos*")) OR "psychosis" OR "psychot*" OR "schizophreni*") AND ("forensi*" OR "*prison*" OR "incarcerat*" OR "jail*" OR "peniten*" OR "convict*" OR "inmate*" OR "detention*" OR "arrest*" OR "detain*")) |
| Filter: ≥ 1990; "Article" |
| EMBASE: 4,825 |
| **No. Query Results**  **#34 #19** AND **#32** AND [1990-2018]/py 4,379  **#33 #19** AND **#32**  4,944  **#32 #22** OR **#27** OR **#30** OR **#31**  432,368  **#31 forensi*** OR **prison*** OR **incarcerat* OR jail*** OR **peniten*** OR **convict*** OR **inmate*** OR **detention*** OR **arrest*** OR **detain***  432,368  **#30 #28** NOT **#29**  15,189  **#29 'prisoner of war'/de**  471  **#28 'prisoner'/exp**  15,660  **#27 #23** NOT **#26**  12,580  **#26 #24** OR **#25**  28,350  **#25** 'insanity defense':ti,kw 263  **#24 'confidentiality'/de** OR **'professional secrecy'/de**  28,087  **#23 'forensic psychiatry'/de**  12,996  **#22 #20** NOT **#21**  14,536  **#21 'concentration camp'/de**  152  **#20 'prison'/exp**  14,688  **#19 #13** AND **#18**  56,960  **#18** #14 0R #15 0R #16 0R #17 510,165  **#17 psychosis** OR **psychot*** OR **schizophreni***  478,221  **#16 severe** NEAR/3 **(mental** OR **psychiatric)** NEAR/3 **(disorder*** OR **illness*** OR **condition*** OR **disease*** OR **diagnos*)**  9,516  **#15 'psychosis'/exp**  278,320  **#14 'schizophrenia spectrum disorder'/exp**  181,063  **#13 #1** OR **#2** OR **#9** OR **#10** OR **#11** OR **#12**  571,697  **#12 suicid***  120,109  **#11 self** NEAR/2 **(harm*** OR **injur*** OR **mutilat*** OR **destruct*)**  16,812  **#10 violen*** OR **aggressi*** OR **agitat*** OR **assault*** OR **homicid***  416,518  **#9 #3** NOT **#8**  76,809  **#8 #4** OR **#5** OR **#6** OR **#7**  62,355  **#7 'terrorism'/exp**  8,503  **#6 'partner violence'/exp**  9,899  **#5 'gender based violence'/de**  420  **#4 'domestic violence'/exp**  53,684  **#3 'violence'/exp**  131,650  **#2 'automutilation'/de**  15,780  **#1 'aggression'/exp**  95,095 |
